# Draft Genome Sequence of the Marine Bacterium *Pseudomonas aestusnigri* VGXO14^T^

**DOI:** 10.1128/genomeA.00765-17

**Published:** 2017-08-10

**Authors:** Margarita Gomila, Magdalena Mulet, Jorge Lalucat, Elena García-Valdés

**Affiliations:** a Departamento de Biología, Microbiología, Universidad de las Islas Baleares, Palma, Illes Balears, Spain; b Institut Mediterrani d’Estudis Avançats, IMEDEA, CSIC, UIB, Palma de Mallorca, Spain

## Abstract

The type strain of *Pseudomonas aestusnigri* (VGXO14), isolated from a crude oil-polluted marine sand sample, is a member of the *P. pertucinogena* phylogenetic group. Here, we report the genome sequence (3.83 Mb) of *P. aestusnigri* to gain insights into the biology and taxonomy of marine *Pseudomonas* spp. adapted to polluted marine habitats.

## GENOME ANNOUNCEMENT

Two main lineages, *Pseudomonas fluorescens* and *P. aeruginosa*, and 11 phylogenetic groups are distinguished in the genus *Pseudomonas* (1). The *P. pertucinogena* group comprises 15 different recognized species, most of them of marine origin. *P. aestusnigri* belongs to the *P. pertucinogena* group (2). All strains so far described within the species have been isolated from marine sand samples contaminated by a crude oil spill (2). To gain insights into the biology of *P. aestusnigri* and to clarify the phylogeny and taxonomy of strains within the group, the whole genome of the type strain, VGXO14 (equivalent to CECT 8317^T^ and CCUG 64165^T^), was sequenced, annotated, and analyzed.

The whole-genome shotgun sequencing of *P. aestusnigri* VGXO14^T^ was performed by using paired-end sequencing with the MiSeq platform (Illumina). The Newbler Assembler version 2.7 software package (Roche) was used for *de novo* genome assembly. The draft genome size is 3,829,769 bp and contains 30 scaffolds, with an average scaffold size of 127,658 bp, a median coverage depth of 75×, and an average G+C content of 60.94 mol%.

The genome prediction and annotation were performed using the NCBI Prokaryotic Genome Annotation Pipeline (http://www.ncbi.nlm.nih.gov/genome/annotation_prok). Analysis and comparison of the functional annotation were done using the KEGG automatic annotation server (3).

Fifty-one RNA genes (44 tRNA genes) were detected, and 3,452 of 3,537 protein-coding genes were annotated. No *Pseudomonas* genome sequence in the publicly available databases showed an ANIb value higher than 90%. No plasmid was found. In addition to genes coding for relevant taxonomic traits of *Pseudomonas* spp. (catalase, superoxide dismutase, flagellation, type IV pilus, twitching motility, catecholate type siderophore, urea catabolism, the synthesis of ectoine as an osmoprotectant—which allows growth at high NaCl concentrations—and the genes required for the synthesis of polyhydroxyalkanoates), strain VGXO14^T^ possessed genetic determinants for adapting to highly polluted environments, including genes related to the resistance to toxic metals (Co, Zn, Cd, and As) as well as those for hydrocarbon degradation. A cluster of genes for the catabolism of monoaromatic hydrocarbons with a multicomponent phenol hydroxylase, a Fis family regulator, and a meta-cleavage pathway of catechol was very similar to the corresponding genes of *P. pachastrellae* LMG 23570^T^ and *P. stutzeri* ST-9 (4). Alkanes might also be degraded through an alkane 1-monooxygenase and a rubredoxin conducting to the ω-hydroxyacid, which is degraded to acetyl-CoA. Toxin-antitoxin systems are considered important for niche-specific colonization (5). A CptA-CptB system was found in the VGXO14^T^ genome, and a complete set of genes for a type VI secretion system was predicted, together with the secreted proteins Hcp and VgrG. The mobilome consisted of at least 29 genes related to prophages and transposons: 11 transposons and 2 integrases. A CRISPR system comprising 6 genes (Csy1, Csy2, Csy3, Csy4, Cas1, and Cas3) was detected.

The genome sequence of *P. aestusnigri* VGXO14^T^ will help in clarifying the taxonomic affiliation of strains within the *P. pertucinogena* group, which includes many species adapted to polluted marine environments.

### Accession number(s).

This whole-genome shotgun project has been deposited at DDBJ/ENA/GenBank under the accession number NBYK00000000. The version described in this paper is the first version, NBYK01000000.
